# Cross-sectional and prospective mediating effects of dietary intake on the relationship between sedentary behaviour and body mass index in adolescents

**DOI:** 10.1186/s12889-017-4771-0

**Published:** 2017-09-29

**Authors:** Elly A. Fletcher, Karen E. Lamb, Sarah A. McNaughton, Sarah P. Garnett, David W. Dunstan, Louise A. Baur, Jo Salmon

**Affiliations:** 1 Deakin University, Geelong, Australia, Institute for Physical Activity and Nutrition (IPAN), School of Exercise and Nutrition Sciences, Geelong, Australia; 2 The Children’s Hospital at Westmead Clinical School, University of Sydney, Westmead, Australia; 3 Baker IDI Heart and Diabetes Institute, Melbourne, VIC Australia; 4 Department of Medicine, Monash University, Melbourne, Australia; 5 The University of Queensland, School of Public Health, Brisbane, Australia; 6 Department of Epidemiology and Preventive Medicine, Monash University, Melbourne, Australia; 7 School of Sport Science, Exercise and Health, The University of Western Australia, Perth, Australia; 8 Mary MacKillop Institute for Health Research, Australian Catholic University, Melbourne, Australia

**Keywords:** Television viewing, Sedentary behaviour, Dietary intake, BMI, Adolescents

## Abstract

**Background:**

Cross-sectional evidence suggests TV viewing, but not objectively-measured sedentary time or bouts of sedentary time, is consistently associated with body mass index (BMI) in adolescents. However, it is unclear whether dietary intake is a potential mediator of these relationships. The aim of this study was to explore the cross-sectional and prospective mediating effects of dietary intake on the association of sedentary behaviour with BMI z-score (zBMI) in a cohort of Australian adolescents.

**Methods:**

Cross-sectional and prospective analyses were conducted in adolescents aged 12–15 years participating in the 2002/03 (baseline) and 2004/05 (follow-up) Nepean Growing Up Study. The independent variables were television (TV) viewing, an objective measure of total sedentary time and average sedentary bout duration, and the outcome variable zBMI. Using the Sobel-Goodman method with bootstrapping, mediation analyses were conducted examining three dietary components (discretionary foods, sugar-sweetened beverages [SSB] and takeaway foods) as mediators of associations between TV viewing and zBMI (*n* = 259) and between total sedentary time and average sedentary bout duration with zBMI (*n* = 140).

**Results:**

No significant cross-sectional or prospective total or direct associations were observed for TV viewing, total sedentary time and average sedentary bout duration with zBMI. However, TV viewing was positively associated with consumption of takeaway foods cross-sectionally (β = 0.06; 95% CI 0.01 to 0.12), prospectively at baseline (β = 0.07; 95% CI 0.01 to 0.12) and prospectively at follow-up (β = 0.10; 95% CI 0.04, 0.16), and average sedentary bout duration was inversely associated with SSB consumption both cross-sectionally (β = −0.36; 95% CI -0.69 to −0.02) and prospectively at baseline (β = −0.36; 95% CI -0.70 to −0.02). No mediation effects were identified.

**Conclusions:**

TV viewing, total sedentary time and bouts of sedentary time were not associated cross-sectionally or prospectively with adolescents’ zBMI, and three elements of dietary intake (e.g. intake of discretionary foods, SSB and takeaway foods) did not mediate this relationship. The role of dietary intake and sedentary behaviour in relation to adolescent health requires further clarification.

**Electronic supplementary material:**

The online version of this article (10.1186/s12889-017-4771-0) contains supplementary material, which is available to authorized users.

## Background

Adolescent obesity is a major public health concern. The combined rates of overweight and obesity among adolescents have increased over the last two decades worldwide [1]. In the United States, the proportion of obese adolescents has risen from 10.5% in 1988–1994 to 20.6% in 2013–2014 [2]. Australia has experienced similar increases with almost one in three adolescents currently overweight or obese [3]. Given that obesity tracks from adolescence to adulthood [4], it is imperative to understand the lifestyle risk factors associated with adolescent obesity, particularly prospectively, in order to inform effective interventions.

Sedentary behaviour – defined as any waking behaviours characterised by low energy expenditure (< 1.5 METS) while in a sitting or reclining posture – has emerged as a new research focus for obesity prevention [5]. High amounts of television (TV) viewing, a common leisure-time sedentary behaviour, during adolescence have both immediate and long-term health consequences, including a higher risk of obesity [6]. However, there are inconsistent associations between total time spent in sedentary time [7, 8], or time spent in periods, or ‘bouts’, of sedentary time [9], and indicators of adiposity (e.g. BMI, waist circumference) in adolescents.

One potential behavioural mechanism that could explain why TV viewing has more consistent associations with body mass index (BMI) when compared to total or bouts of sedentary time in adolescents, is an increase in energy-dense, nutrient poor foods and sugar-sweetened beverages (SSB). For example, TV viewing has consistently been reported to be associated with a higher energy intake, and an increased consumption of discretionary foods, SSB, and fast food/takeaway foods in adolescents [10], whereas few studies have reported associations between objectively-measures of sedentary time with dietary intake [11, 12]. In addition, no study to date has explored whether prolonged bouts of sedentary time are related to dietary intake among adolescents. The latter is important as studies with adults have shown that, independent of how much total sedentary time is accumulated, those with fewer interruptions in sedentary time (i.e., prolonged bouts) have poorer cardiometabolic health profiles [13].

A systematic review examining whether associations between sedentary behaviour and health outcomes in adolescents were independent of dietary intake found TV viewing, screen time and overall sedentary time were positively related to BMI, independent of dietary intake [14]. The systematic review also identified very few studies had specifically examined the mediating role of dietary intake in the TV viewing and BMI relation; with only two studies (out of the 21 studies identified) exploring this and reporting no mediation effects [15, 16]. However, a major limitation of both of these studies was their cross-sectional design which limits causal inference. Whereas, a longitudinal design would allow both the temporal order of associations to be examined and many other aspects of a mediation model to be explored.

Against this background, the primary aim of the study was to explore both the cross-sectional and prospective mediating effects of the consumption of discretionary foods, SSB and takeaway foods on the association between TV viewing and BMI z-score (zBMI) in a cohort of Australian adolescents, and to examine whether these findings differ when total sedentary time and sedentary bout duration are examined. The secondary aims of the study were to explore the individual associations between the sedentary behaviour variables (TV viewing, sedentary time and sedentary bout duration) and dietary intake variables (discretionary foods, SSB and takeaway foods), as well as their associations with zBMI. Based on the existing evidence, we hypothesised that consumption of discretionary foods, SSB and takeaway foods would partially mediate the cross-sectional and prospective association between TV viewing and zBMI, but would not mediate the cross-sectional or prospective association between total sedentary time and sedentary bouts with zBMI.

## Methods

### Study design

In 2002/03 (baseline), 348 adolescents aged 12–13 years participated in the Nepean Kids Growing-Up Study. The adolescents were originally part of a birth cohort study (the “Nepean Study”) which involved 2314 infants born between 1989 and 1990 at the Nepean Hospital (western Sydney, Australia). Written consent was obtained from the parent or guardian and assent from the adolescent. The Ethics Committees of The Children’s Hospital at Westmead and Wentworth Area Health Service gave ethical approval. Full details about the original study and eligibility criteria have been previously published [17]. Briefly, the study involved adolescents attending the clinic at Nepean Hospital, where they had their height and weight measured and completed a questionnaire on their demographics and physical activity levels, and a semi-quantitative food frequency questionnaire (FFQ). Afterwards, adolescents wore an accelerometer for 7 days during all waking hours. In 2004/05, the adolescents were recontacted and invited to participate in the follow-up study. In total, 63 adolescents were unable to be contacted or withdrew from the study, leaving 285 adolescents participating at both time points.

### Outcome variable (zBMI)

Height and weight were measured at both time points by a trained research assistant and study dietitian at the clinic. Height was measured to the nearest 0.1 cm and weight was measured without shoes and in light clothing to the nearest 0.1 kg with electronic scales (Wedderburn, Summer Hill, NSW, Australia). Height and weight were used to calculate each participants’ BMI and zBMI was determined using the age- and sex-specific CDC 2000 reference data [18]. Overweight and obesity was determined using the International Obesity Task Force (IOTF) criteria [19]. In all analyses, zBMI was treated as a continuous variable.

### Independent variables (sedentary behaviour)

Adolescents completed a self-report questionnaire on their time spent watching TV (hours/day) on a usual school day (Monday to Friday) and a usual weekend day (Saturday and Sunday). The questionnaire has previously been shown to have good to excellent reliability (percentage agreement = 70%–99%) [20]. To calculate average daily hours spent watching TV over a usual week, daily weekday TV hours was multiplied by five and daily weekend TV hours was multiplied by 2, then summed together and divided by seven.

Sedentary time was measured objectively by an ActiGraph AM-7164 accelerometer (ActiGraph Inc., Florida). At both time points, adolescents were asked to wear the monitor on their right hip during all waking hours for 7 days, except when bathing, swimming and sleeping. Data were downloaded in 1-min epochs and non-wear time was defined as at least 20 min of zero counts. Sedentary time was defined as all wear-time minutes with an average activity count of ≤100 counts per minute (cpm), and was standardised for wear time using the residual method [21]. Average sedentary bout duration was calculated by summing all uninterrupted minutes ≤100 cpm, and then taking the midpoint of all sedentary bouts that lie on the accumulation curve for each individual [22]. Analyses were limited to participants who had ≥8 h of wear time on ≥3 week days and ≥7 h of wear time on ≥1 weekend day [23].

### Mediating variables (dietary intake)

Usual dietary intake was measured using a 56-item semi-quantitative FFQ, which was developed based on data from the 1995 Australian National Nutrition Survey [24]. Adolescents were asked to report how often they ate certain foods and beverages (either in times per week or per day) over the previous 7 days. The 8-item frequency response scale was converted to times per week as follows: 1) “not consumed last week” = 0; 2) “consumed once last week” = 0.143; 3) “consumed 2–3 times last week” (average number used) = 0.357; 4) “consumed 4–6 times last week” (average number used) = 0.714; 5) “consumed once a day” = 1; 6) “consumed 2 times a day” = 2; 7) “consumed 3 times a day” = 3; 8) “consumed 4–6 times a day” (average number used) = 5. For the analyses, a combination of food and beverage items were summed together to create three dietary mediators at both time points: 1) frequency of consuming discretionary foods, which included any savoury or plain biscuits, sweet pastries, cakes, doughnuts, chocolate, confectionary, and potato chips; 2) frequency of consuming SSB, which included non-diet soft drink, non-diet cordial and fruit juice and not sweetened milk drinks or energy drinks; and 3) frequency of consuming takeaway foods, which included savoury pastries (e.g. meat pies and sausage rolls), hamburgers, pizza, hot chips and spring rolls/dim sims. The FFQ was tested for reproducibility and overall showed fair to excellent reliability for sweet snacks (ICC = 0.61), savoury snacks (ICC = 0.63), SSBs (ICC = 0.77), and fast food (ICC = 0.44). Frequency of consuming discretionary foods and frequency of consuming SSB were multiplied by 7 to convert to times per day. Participants with missing data for any of the dietary items listed were excluded (*n* = 2). In all analyses, the dietary mediators were treated as continuous variables.

### Covariates

The covariates considered for the analyses included age at baseline, sex, maternal education (an indicator for family socioeconomic status), pubertal status and accelerometry-measured moderate-to-vigorous physical activity (MVPA) collected at baseline. Maternal education was collapsed into three categories: “low” (some secondary education or less); “medium” (completing secondary school, an apprenticeship or technical certificate); and “high” (university or tertiary qualification). Pubertal status was self-assessed using the ‘Tanner Stages’ of breast development and commencement of menses (girls) and pubic hair and genitalia (boys) [25]. For analyses, participants were categorised as early puberty, mid-pubertal, late-pubertal and post-pubertal. Accelerometry-measured MVPA at baseline was calculated based on the Freedson accelerometer age-cut points [26] and considered as a covariate for the analyses involving sedentary time and sedentary bouts with zBMI.

### Statistical analyses

**Fig. 1:**
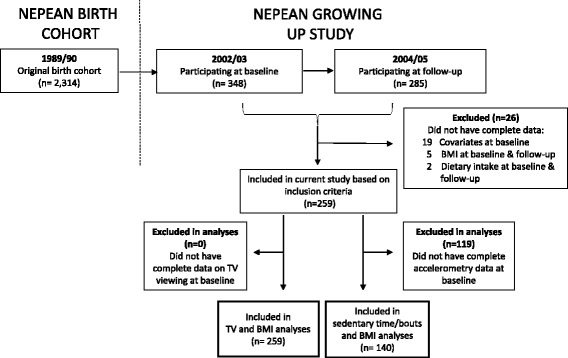
Flow diagram of participants for analyses

As shown in Fig. 1, to be included in the analyses involving TV viewing and zBMI, participants were required to have complete data for age, sex, maternal education, pubertal status, dietary intake and TV viewing at baseline, and complete data for zBMI at baseline and follow-up (*n* = 259). To examine the association of total and bouts of sedentary time, dietary intake and zBMI, separate analyses were undertaken from a subsample of participants who met the previous inclusion criteria as well as meeting the accelerometry inclusion criteria at baseline (*n* = 140). Prior to the main analyses, all variables were checked for normality. Discretionary foods, SSB and takeaway food intake at baseline were not normally distributed and were log-transformed.

**Fig. 2:**
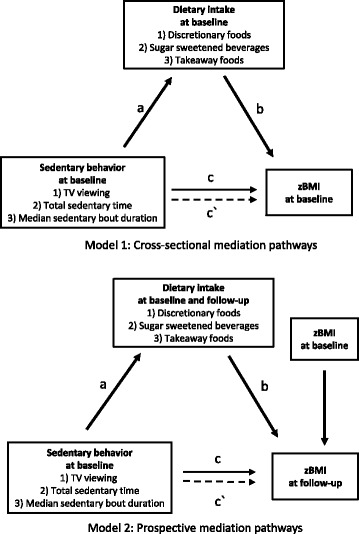
Theoretical diagram of the cross-sectional (Model 1) and prospective (Model 2) mediation pathway

Analyses were conducted using Stata/SE v14.0 (StataCorp LP, College Station, Texas, 2015). Figure 2 illustrates two theoretical models of the cross-sectional and prospective mediation pathways examined [27]. For all mediation analyses, the Sobel-Goodman mediation method with bootstrapping with 5000 replications was used to estimate standard errors and 95% confidence intervals [28]. For the cross-sectional analyses, only baseline variables were used to test the following associations: 1) association between the independent variable and the mediator (a-coefficient pathway); 2) association between the mediator and the outcome variable, adjusting for the independent variable (b-coefficient pathway); 3) total association between the independent variable and outcome variable (c-coefficient pathway); 4) direct association between the independent variable and outcome variable, accounting for each mediator (c′-coefficient pathway); and 5) indirect association (e.g. mediating effect) of the mediator on the independent variable and outcome variable. For the prospective analyses, similar pathways were tested. However, dietary intake at baseline and dietary intake at follow-up were examined separately as potential mediators in the associations between the independent variable at baseline and the outcome variable at follow-up. All analyses were adjusted for age, sex, pubertal status and maternal education, with objectively-measured MVPA adjusted for in the analyses involving total and bouts of sedentary time with zBMI. The prospective analyses were additionally adjusted for zBMI at baseline. The significance level was set at *p* < 0.05 for all statistical tests.

## Results

Overall, 259 and 140 adolescents were included in the TV viewing and zBMI, and the sedentary time and zBMI analytic samples, respectively (Table 1). Those excluded in the TV viewing and zBMI analyses had mothers with a lower maternal education and those excluded in the sedentary time and zBMI analyses were older, had a lower maternal education and had a higher proportion of overweight participants (Additional file 1: Tables S1 and S2 in the online supplement file).

**Table 1:** Baseline characteristics of participants included in analyses

Variables	TV and zBMI (*n* = 259)	Sedentary time and zBMI (*n* = 140)
Age, years	12.9 (12.9, 13.0)	12.9 (12.9, 13.0)
Sex, %		
Male	47.5 (41.4, 53.6)	50.0 (41.7, 58.3)
Female	52.5 (46.4, 58.4)	50.0 (41.7, 58.3)
Maternal education, %		
Low	6.9 (4.4, 10.8)	5.0 (2.3, 10.2)
Medium	65.3 (59.2, 70.8)	62.8 (54.4, 70.5)
High	27.8 (22.7, 33.6)	32.1 (24.8, 40.4)
Pubertal status, %		
Early puberty	19.3 (8.0, 4.6)	14.3 (35.1, 56.2)
Mid-pubertal	35.5 (30.0, 41.6)	41.4 (33.5, 50.0)
Late-pubertal	35.5 (30.0, 41.6)	35.0 (27.5, 43.4)
Post-pubertal	27.0 (21.9, 32.8)	22.1 (16.0, 30.0)
BMI		
kg/m^2^	20.7 (20.2, 21.3)	20.3 (19.7, 21.0)
z-score	0.4 (0.3, 0.5)	0.3 (0.1, 0.5)
Overweight, %	18.9 (14.6, 24.2)	13.6 (8.8, 20.4)
Obese, %	9.2 (6.3, 13.5)	9.2 (5.4, 15.4)
Sedentary behaviour		
TV viewing (hours/day)	3.2 (3.0, 3.4)	
Total sedentary time (hours/day)		6.1 (6.0, 6.2)
Average sedentary bout (minutes/day)		6.6 (6.5, 6.6)
MVPA (minutes/day)		48.2 (35.0, 61.4)
Dietary intake		
Discretionary foods (freq/day)	2.4 (2.2, 2.6)	2.4 (2.1, 2.7)
Sugar-sweetened beverages (freq/day)	2.1 (1.8, 2.3)	1.9 (1.6, 2.2)
Takeaway food (freq/week)	3.4 (3.1, 3.6)	3.2 (2.8, 3.6)

*BMI* body mass index, *zBMI* BMI z-score, *TV* television, *MVPA* moderate-to-vigorous physical activity; freq: frequency

Footnote: Maternal education: low = some secondary education or less; medium = completing secondary school, an apprenticeship or technical certificate; high = university or tertiary qualification. Pubertal status self-assessed using the ‘Tanner Stages’ [25]. BMI z-score determined using the age- and sex-specific CDC 2000 reference data [18]. Overweight and obesity determined using the International Obesity Task Force (IOTF) criteria [19]

### Associations between TV viewing, dietary intake and zBMI

#### Cross-sectionally

There was no evidence of total or direct cross-sectional associations of TV viewing with zBMI (Table 2). A small, positive association was observed between hours spent watching TV per day and frequency of consuming takeaway foods each week (a-coefficient pathway); with each additional hour of TV viewing, adolescents consumed an additional 0.06 serves (95% CI 0.01 to 0.12; *p* < 0.05) of takeaway foods each week. An inverse association was also observed for consuming discretionary foods each day and zBMI (b-coefficient pathway); with each additional serving of discretionary foods consumed each day, zBMI was lower by −0.39 units (95% CI -0.65 to −0.13; *p* < 0.01). None of the dietary variables were significant mediators of the cross-sectional association of TV viewing with zBMI.

**Table 2:** Cross-sectional^a^ and prospective^b^ associations of dietary intake (mediator), TV viewing (independent) and zBMI (outcome) (*n* = 259)

Outcome: zBMI	c`-coefficient (direct) β (95% CI)	a-coefficient β (95% CI)	b-coefficient β (95% CI)	ab/indirect (mediated/indirect)^c^	
Independent: TV viewing				Uncorrected β (95% CI)	Bias-corrected β (95% CI)
Cross-sectional mediators					
Discretionary food (freq/day)	0.05 (−0.02, 0.13)	0.01 (−0.03, 0.05)	−0.39 (−0.65, −0.13)**	−0.04 (−0.18, 0.10)	−0.04 (−0.23, 0.12)
SSB (freq/day)	0.05 (−0.03, 0.13)	0.02 (−0.03, 0.07)	0.03 (−0.15, 0.22)	0.01 (−0.04, 0.05)	0.01 (−0.07, 0.09)
Takeaway food (freq/week)	0.05 (−0.02, 0.13)	0.06 (0.01, 0.12)*	−0.07 (−0.24, 0.09)	−0.05 (−0.16, 0.07)	−0.05 (−0.22, 0.06)
Prospective mediators					
At baseline					
Discretionary foods (freq/day)	−0.01 (−0.04, 0.02)	0.01 (−0.02, 0.05)	0.02 (−0.09, 0.13)	0.01 (−0.01, 0.02)	0.01 (−0.02, 0.04)
SSB (freq/day)	−0.01 (−0.04, 0.02)	0.02 (−0.03, 0.07)	0.03 (−0.04, 0.11)	0.01 (−0.02, 0.03)	0.01 (−0.02, 0.04)
Takeaway food (freq/week)	−0.01 (−0.04, 0.02)	0.07 (0.01, 0.12)*	−0.01 (−0.07, 0.06)	−0.01 (−0.04, 0.05)	−0.01 (−0.06, 0.05)
At follow-up					
Discretionary foods (freq/day)	−0.01 (−0.04, 0.02)	0.02 (−0.02, 0.07)	−0.04 (−0.13, 0.06)	−0.01 (−0.04, 0.02)	−0.01 (−0.06, 0.02)
SSB (freq/day)	−0.01 (−0.04, 0.02)	−0.01 (−0.05, 0.04)	0.03 (−0.06, 0.11)	−0.001 (−0.01, 0.01)	−0.001 (−0.03, 0.02)
Takeaway food (freq/week)	−0.01 (−0.04, 0.02)	0.10 (0.04, 0.16)*	0.02 (−0.05, 0.08)	0.02 (−0.05, 0.08)	0.02 (−0.04, 0.09)

^a^Cross-sectional total effects (c-pathway) of TV viewing and zBMI β = 0.05 (95% CI -0.03 to 0.13), adjusting for age, sex, mother’s education and pubertal status. ^b^Prospective total effects (c-pathway) of TV viewing and zBMI β = −0.01 (95% CI -0.04 to 0.02), adjusting for age, sex, mother’s education, pubertal status and zBMI at baseline. ^c^Due to the small units of measure, the indirect effects have been multiplied by 10

Significant **p < 0.01, *p < 0.05. TV: television; SSB: sugar-sweetened beverages; zBMI: body mass index z-score; freq: frequency

#### Prospectively

There was no evidence of total or direct prospective associations between TV viewing at baseline and zBMI at follow-up. Similar to the cross-sectional associations, a positive prospective association was observed between hours spent watching TV per day and frequency of consuming takeaway foods each week at baseline and at follow-up. For example, for each additional hour of TV viewing, adolescents consumed an additional 0.07 (95% CI 0.01 to 0.12; *p* < 0.05) serves of take-away foods each week at baseline, and an additional 0.10 (95% CI 0.04 to 0.16; *p* < 0.05) serves of take-away foods each week at follow-up. However, no significant association remained for any of the dietary variables consumed at baseline and at follow-up with zBMI at follow-up (b-coefficient pathway). None of the dietary variables were significant mediators of the prospective association between TV viewing and zBMI.

### Associations between total sedentary time, dietary intake and zBMI

#### Cross-sectionally

There was no evidence of total or direct cross-sectional associations for total sedentary time (hours/day) and zBMI, or between total sedentary time and any of the dietary mediators (a-coefficient pathway) (Table 3). An inverse association was observed between frequency of consuming discretionary foods per day and zBMI; with each additional discretionary food consumed each week, zBMI was lower by −0.42 units (95% CI -0.77 to −0.07; *p* < 0.05) (b-coefficient). There were no significant mediating effects for any of the dietary variables in the cross-sectional association of total sedentary time with zBMI.

**Table 3:** Cross-sectional^a^ and prospective^b^ associations of dietary intake (mediator), sedentary time (independent) and zBMI (outcome) (*n* = 140)

Outcome: zBMI	c`-coefficient (direct) β (95% CI)	a-coefficient β (95% CI)	b-coefficient β (95% CI)	ab/indirect (mediated/indirect)^c^	
Independent: total sedentary time				Uncorrected β (95% CI)	Bias-corrected β (SE)
Cross-sectional mediators					
Discretionary food (freq/day)	0.27 (−0.03, 0.58)	−0.01 (−0.15, 0.14)	−0.42 (−0.77, −0.07)*	0.01 (−0.61, 0.62)	0.01 (−0.62, 0.69)
SSB (freq/day)	0.27 (−0.05, 0.58)	−0.16 (−0.38, 0.06)	−0.04 (−0.29, 0.20)	0.07 (−0.33, 0.46)	0.07 (−0.31, 0.61)
Takeaway food (freq/week)	0.27 (−0.05, 0.58)	−0.06 (−0.30, 0.18)	−0.04 (−0.26, 0.18)	0.02 (−0.13, 0.18)	0.02 (−0.36, 0.35)
Prospective mediators					
At baseline					
Discretionary food (freq/day)	0.05 (−0.07, 0.17)	0.03 (−0.12, 0.17)	0.03 (−0.10, 0.17)	0.01 (−0.05, 0.07)	0.01 (−0.08, 0.15)
SSB (freq/day)	0.05 (−0.06, 0.17)	−0.15 (−0.37, 0.07)	0.02 (−0.07, 0.11)	−0.03 (−0.17, 0.11)	−0.03 (−0.22, 0.13)
Takeaway food (freq/week)	0.05 (−0.07, 0.17)	−0.05 (−0.30, 0.19)	−0.03 (−0.12, 0.05)	0.02 (−0.08, 0.11)	0.02 (−0.12, 0.16)
At follow-up					
Discretionary food (freq/day)	0.05 (−0.07, 0.17)	0.08 (−0.09, 0.24)	0.01 (−0.11, 0.13)	0.01 (−0.05, 0.10)	0.01 (−0.16, 0.17)
SSB (freq/day)	0.05 (−0.06, 0.17)	−0.04 (−0.23, 0.15)	0.01 (−0.10, 0.12)	−0.01 (−0.05, 0.04)	−0.01 (−0.11, 0.09)
Takeaway food (freq/week)	0.05 (−0.06, 0.17)	0.21 (−0.57, 0.47)	−0.01 (−0.08, 0.07)	−0.01 (−0.16, 0.15)	−0.01 (−0.19, 0.17)

^a^Cross-sectional total effects (c-pathway) of total sedentary time and zBMI β = 0.27 (95% CI -0.04 to 0.58), adjusting for age, sex, mother’s education and pubertal status. ^b^Prospective total effects (c-pathway) of total sedentary time and zBMI β = 0.05 (95% CI -0.06 to 0.17), adjusting for age, sex, mother’s education, pubertal status and zBMI at baseline. ^c^Due to the small units of measure, the indirect effects have been multiplied by 10. Significant *p < 0.05. SSB: sugar-sweetened beverages; zBMI: body mass index z-score; freq: frequency

#### Prospectively

Total sedentary time at baseline was not significantly associated with zBMI at follow-up, accounting for mediation by dietary intake at baseline and at follow-up. No significant associations were also observed for any of the total, direct and indirect pathways, nor with the a-coefficient and b-coefficient pathways.

### Associations between average sedentary bout duration, dietary intake and zBMI

#### Cross-sectionally

No significant total or direct cross-sectional associations were observed for average sedentary bout duration (minutes/day) with zBMI (Table 4). Average sedentary bout duration was inversely related to frequency of consuming SSB each day (a-coefficient pathway); with each additional minute spent in a sedentary bout, the frequency of consuming SSB was lower by nearly half a serve each day (β = −0.36, 95% CI -0.69 to −0.02; *p* < 0.05). An inverse association was also observed for frequency of consuming discretionary foods each week and zBMI (b-coefficient pathway); with each additional discretionary food consumed each week, zBMI was lower by −0.42 units (95% CI -0.77 to −0.06; *p* < 0.05). None of the dietary variables significantly mediated cross-sectional associations of sedentary bout duration with zBMI.

**Table 4:** Cross-sectional^a^ and prospective^b^ associations of dietary intake (mediator), sedentary bouts (independent) and zBMI (outcome) (*n* = 140)

Outcome: zBMI	c`-coefficient (direct) β (95% CI)	a-coefficient β (95% CI)	b-coefficient β (95% CI)	ab/indirect (mediated/indirect)^c^	
Independent: sedentary bouts				Uncorrected β (95% CI)	Bias-corrected β (SE)
Cross-sectional mediators					
Discretionary food (freq/day)	0.12 (−0.36, 0.61)	−0.08 (−0.31, 0.15)	−0.42 (−0.77, −0.06)*	0.32 (−0.67, 1.32)	0.32 (−0.55, 1.37)
SSB (freq/day)	0.14 (−0.36, 0.64)	−0.36 (−0.69, −0.02)*	−0.06 (−0.30, 0.19)	0.20 (−0.71, 1.11)	0.20 (−0.68, 0.12)
Takeaway food (freq/week)	0.16 (−0.33, 0.65)	0.03 (−0.34, 0.41)	−0.05 (−0.28, 0.18)	−0.02 (−0.21, 0.18)	−0.02 (−0.62, 0.34)
Prospective mediators					
At baseline					
Discretionary food (freq/day)	0.08 (−0.10, 0.26)	−0.06 (−0.29, 0.17)	0.04 (−0.10, 0.17)	−0.02 (−0.14, 0.10)	−0.02 (−0.24, 0.14)
SSB (freq/day)	0.09 (−0.09, 0.27)	−0.36 (−0.70, −0.02)*	0.02 (−0.07, 0.11)	−0.09 (−0.42, 0.24)	−0.09 (−0.52, 0.24)
Takeaway food (freq/week)	0.08 (−0.10, 0.26)	0.04 (−0.34, 0.41)	−0.04 (−0.12, 0.04)	−0.01 (−0.15, 0.13)	−0.01 (−0.27, 0.16)
At follow-up					
Discretionary food (freq/day)	0.08 (−0.10, 0.26)	−0.08 (−0.34, 0.17)	0.02 (−0.10, 0.14)	−0.02 (−0.12, 0.09)	−0.02 (−0.28, 0.16)
SSB (freq/day)	0.08 (−0.10, 0.26)	0.11 (−0.18, 0.40)	0.01 (−0.010, 0.11)	0.01 (−0.11, 0.12)	0.01 (−0.19, 0.20)
Takeaway food (freq/week)	0.08 (−0.10, 0.26)	0.06 (−0.35, 0.48)	−0.01 (−0.08, 0.07)	−0.01 (−0.05, 0.04)	−0.01 (0.18, 0.17)

^a^Cross-sectional total effects (c-pathway) of average sedentary bout and zBMI β = 0.16 (95% CI -0.33 to 0.65), adjusting for age, sex, mother’s education and pubertal status. ^b^ Prospective total effects (c-pathway) of average sedentary bout and zBMI β = 0.08 (95% CI -0.10 to 0.26), adjusting for age, sex, mother’s education, pubertal status and zBMI at baseline. ^c^Due to the small units of measure, the indirect effects have been multiplied by 10. Significant *p < 0.05. SSB: sugar-sweetened beverages; zBMI: body mass index z-score; freq: frequency

#### Prospectively

When examining the prospective associations between average sedentary bout duration at baseline and zBMI at follow-up, no significant total or direct associations were observed. However, a significant inverse association remained for average sedentary bout duration with frequency of consuming SSB at baseline (β = −0.36; 95% CI -0.70 to −0.02; *p* < 0.05), but not at follow-up for the a-coefficient pathways. No significant associations were observed for the b-coefficient pathways or mediating effects.

## Discussion

This study found no evidence of direct or indirect associations for TV viewing, total sedentary time and average sedentary bout duration with adolescents’ zBMI, either cross-sectionally or prospectively. Although some of the dietary variables were independently associated with TV viewing, average sedentary bout duration and zBMI, none of the dietary variables significantly mediated the relationships between the sedentary variables and zBMI cross-sectionally or prospectively.

The null finding for the association of TV viewing with zBMI in the current study is in contrast to previous studies in youth that have consistently shown significant and positive associations both cross-sectionally [6] and prospectively [29, 30]. The differences in findings could be attributed to the homogeneity of the current sample being examined, with higher than average number of hours spent watching TV [31] and a lower zBMI compared to the population average [32]. The null association for total sedentary time and average sedentary bout duration with zBMI is supported by some previous studies [8, 33–35], but not others [7]. Research examining accelerometer-measured sedentary time with health indictors among children and youth appears to be mixed; it is unclear whether an association exists in only some populations or if there are inconsistencies in measuring sedentary time and/or the analytical approaches undertaken.

The positive association observed for TV viewing with the consumption of takeaway foods, both cross-sectionally and prospectively is consistent with previous research [36, 37]. This link could be partially explained by the large extent of TV advertising of foods high in fat and energy during peak times when children and adolescents are likely to be watching TV [38]. In contrast, no evidence of an association was observed for total sedentary time with any of the dietary variables. The null finding is consistent with previous literature in youth [11, 12] where, compared to TV viewing, fewer significant associations are observed for total sedentary time with elements of a less healthy diet. The null finding could be due to the measure used to capture total sedentary time. For example, accelerometers measure *all* time spent being sedentary, and thus may capture times where adolescents may not be eating/drinking (e.g. sitting in school, sitting in the car). In addition, due to accelerometers being unable to determine posture (e.g. standing still versus sitting), time spent standing may have been included in total sedentary time and thus may diminish the opportunity to engage in an eating occasion. Unexpectedly, the study found higher sedentary bout duration was inversely associated with a lower consumption of SSB, both cross-sectionally and prospectively. One possible reason for this could be due to the adolescents not breaking up their sedentary time in order to retrieve a SSB from another room (e.g. the kitchen, school canteen). However, given the current study is one of the first to examine individual associations between sedentary bout duration and dietary intake, further research is urgently needed in this area.

In contrast, the cross-sectional, inverse association found for discretionary foods with zBMI, and the null finding for SSB and takeaway foods is in contrast with previous studies that have found positive associations for unhealthy dietary intake with BMI [39, 40]. Our findings could be a consequence of under-reporting, where overweight or obese children and adolescents have been found to under-report their energy intake by 20–40% [41]. Alternatively, it is possible that some participants in the current study with a higher zBMI may have changed their behaviour by decreasing their discretionary food intake over time as a strategy to manage their weight.

Only one other study has explored the prospective associations between TV viewing, dietary intake and BMI; however, that study examined a younger population of pre-school children aged 0–5 years over a 2-year period [42]. In contrast to the current study, Fuller-Tyszkiewicz et al. reported a significant positive association between TV viewing and BMI that was bi-directional, with those children characterised with high amounts of TV viewing having higher BMI, and children with higher BMI watching a greater amount of TV. In addition, that study reported the prospective associations between TV viewing and BMI among 4 year olds were mediated by discretionary foods and soft drink consumption [42]. The differences in findings between the current study and previous study could be contributed to the different study populations and dietary mediators being examined, and that the previous study only examined TV viewing and BMI. Thus, further prospective studies are needed to explore whether dietary intake mediates the relationship between various sedentary behaviours (both subjective and objectively measured) and health indicators (e.g. BMI, metabolic syndrome).

To our knowledge, the current study is the first to examine both the cross-sectional and prospective mediating effects of dietary intake on the association between sedentary behaviour and zBMI in an adolescent population, and the first to examine this using objective measures of sedentary time. Other strengths include adjustment for a variety of confounders, including maternal education and adolescent pubertal status, and examining the dietary mediators (discretionary foods, SSB, takeaway foods) separately in all models. Limitations of the study include participants self-reporting their dietary intake using an FFQ and hours spent watching TV, and the low sample size for mediation analyses. In addition, the semi-quantitative FFQ used in the current study was limited with the number of healthy food items included (e.g. fruit and vegetables) and did not have information on portion sizes. There were also differences in those that were included and excluded in the analysis of this study and thus may limit the representative of the findings. Further, the data presented in the current study was collected more than a decade ago and thus the behaviours reported in the study may not reflect the contemporary sedentary and dietary behaviours adolescents are engaging in today.

## Conclusion

In conclusion, despite identifying some significant associations between TV viewing and average sedentary bout duration with frequency of consuming takeaway foods and SSB, and between frequency of consuming discretionary foods and zBMI, no significant associations were observed for any of the sedentary behaviour variables with zBMI, either cross-sectionally or prospectively. In addition, none of the dietary variables were found to be significant mediators of the associations between sedentary behaviour and zBMI. Given the unacceptably high levels of adolescent overweight and obesity, further studies are warranted to elucidate the complex relationships between TV viewing, sedentary time, dietary intake and health indicators.

## Additional file

Additional file 1: Supplement file. Comparison of baseline characteristics of participants included in the TV viewing and zBMI analyses compared to those excluded in the analyses. (PDF 29 kb)

## Abbreviations

BMI: Body mass index
Cpm: Counts per minute
FFQ: Food frequency questionnaire
MVPA: Moderate-to-vigorous physical activity
SSB: Sugar-sweetened beverages
TV: Television
zBMI: Body mass index z-score
