# Supplementary material for: Cross-sectional and prospective mediating effects of dietary intake on the relationship between sedentary behaviour and body mass index in adolescents
Source: BMC Public Health. 2017 Sep 29;17:751. doi: 10.1186/s12889-017-4771-0 (PMC5622455; doi:10.1186/s12889-017-4771-0)
Supplement: Additional file 1: — Supplement file. Comparison of baseline characteristics of participants included in the TV viewing and zBMI analyses compared to those excluded in the analyses. (PDF 29 kb) [file 12889_2017_4771_MOESM1_ESM.pdf]

ADDITIONAL FILE 1

Cross-sectional and prospective mediating effects of dietary intake on the relationship between sedentary behaviour and body mass index in adolescents

Elly A. Fletcher[1], Karen E. Lamb[1], Sarah A. McNaughton[1], Sarah P. Garnett[2], David W. Dunstan[1, 3-8], Louise A. Baur[2], Jo Salmon[1]

[1]Deakin University, Geelong, Australia, Institute for Physical Activity and Nutrition (IPAN), School of Exercise and Nutrition Sciences. [2]The Children's Hospital at Westmead Clinical School, University of Sydney, Westmead, Australia. [3]Baker IDI Heart and Diabetes Institute, Melbourne, Victoria, Australia. [4]Department of Medicine, Monash University, Melbourne, Australia. [5]The University of Queensland, School of Public Health, Brisbane, Australia. [6]Department of Epidemiology and Preventive Medicine, Monash University, Melbourne, Australia. [7]School of Sport Science, Exercise and Health, The University of Western Australia, Perth Australia. [8]Mary MacKillop Institute for Health Research, Australian Catholic University, Melbourne Australia.

Corresponding Author

Elly Fletcher, Institute for Physical Activity and Nutrition (IPAN), School of Exercise and Nutrition Sciences, Deakin University, 221 Burwood Highway, Burwood Vic 3125, Australia. Telephone: +61 3 92517262, Email elly.fletcher@deakin.edu.au

Table S1: Comparison of baseline characteristics of participants included in the TV viewing and zBMI analyses (n=259) compared to those excluded in the analyses (n=26)

**Variables**	**Included (n= 259)**	**Excluded (n= 26)**
**Age, years**	12.9 (12.9, 13.0)	13.0 (12.9, 13.0)
**Sex, %**		
Male	47.5 (41.4, 53.6)	56.2 (45.6, 66.3)
Female	52.5 (46.4, 58.4)	43.8 (33.7, 54.5)
**Maternal education, %**		
Low	6.9 (4.4, 10.8)	18.6 (11.6, 28.5)*
Medium	65.3 (59.2, 70.8)	64.0 (53.1, 73.5)
High	27.8 (22.7, 33.6)	17.4 (10.7, 27.2)
**Pubertal status, %**		
Early puberty	19.3 (8.0, 4.6)	0.0 (0.0, 0.0)
Mid-pubertal	35.5 (30.0, 41.6)	31.4 (21.5, 43.5)
Late-pubertal	35.5 (30.0, 41.6)	44.3 (32.9, 56.3)
Post-pubertal	27.0 (21.9, 32.8)	24.3 (15.5, 36.0)
**BMI**		
kg/m^2	20.7 (20.2, 21.3)	20.8 (19.9, 21.7)
z-score	0.4 (0.3, 0.5)	0.4 (0.2, 0.6)
Overweight, %	18.9 (14.6, 24.2)	23.9 (56.4, 76.2)
Obese, %	9.2 (6.3, 13.5)	9.1 (4.5, 17.3)
**Sedentary behaviour**		
TV viewing (hours/day)	3.2 (3.0, 3.4)	3.0 (2.7, 3.3)
**Dietary intake**		
Discretionary foods (freq/day)	2.4 (2.2, 2.6)	2.3 (2.0, 2.7)
Sugar-sweetened beverages (freq/day)	2.1 (1.8, 2.3)	2.0 (14.6, 2.4)
Takeaway food (freq/week)	3.4 (3.1, 3.6)	3.5 (2.9, 4.1)

BMI: Body mass index; zBMI: BMI z-score; TV: television; MVPA: moderate-to-vigorous physical activity; freq: frequency.

*Significant $p < 0.05$.

Footnote: Maternal education: low = some secondary education or less; medium = completing secondary school, an apprenticeship or technical certificate; high = university or tertiary qualification. Pubertal status self-assessed using the ‘Tanner Stages’ [24]. BMI z-score determined using the age- and sex-specific CDC 2000 reference data [18]. Overweight and obesity determined using the International Obesity Task Force (IOTF) criteria [19].

Table S2: Comparison of baseline characteristics of participants included in the sedentary time and zBMI analyses (n=140) compared to those excluded in the analyses (n=145)

**Variables**	**Included (n= 140)**	**Excluded (n= 145)**
**Age, years**	12.9 (12.9, 13.0)	13.0 (12.9, 13.0)*
**Sex, %**		
Male	50.0 (41.7, 58.3)	49.5 (42.7, 56.3)
Female	50.0 (41.7, 58.3)	50.5 (43.7, 57.3)
**Maternal education, %**		
Low	5.0 (2.3, 10.2)	13.2 (9.2, 18.6)*
Medium	62.8 (54.4, 70.5)	66.3 (59.5, 72.5)
High	32.1 (24.8, 40.4)	20.5 (15.5, 26.6)
**Pubertal status, %**		
Early puberty	14.3 (35.1, 56.2)	1.6 (0.5, 4.8)
Mid-pubertal	41.4 (33.5, 50.0)	29.6 (23.5, 36.6)
Late-pubertal	35.0 (27.5, 43.4)	39.2 (32.4, 46.4)
Post-pubertal	22.1 (16.0, 30.0)	29.6 (23.5, 36.6)
**BMI**		
kg/m^2	20.3 (19.7, 21.0)	21.0 (20.4, 21.6)
z-score	0.3 (0.1, 0.5)	0.5 (0.3, 0.6)
Overweight, %	13.6 (8.8, 20.4)	24.6 (19.2, 31.0)*
Obese, %	9.2 (5.4, 15.4)	9.2 (5.9, 14.0)
**Sedentary behaviour**		
Total sedentary time (hours/day)	6.1 (6.0, 6.2)	5.8 (5.3, 6.2)
Average sedentary bouts (minutes/day)	6.6 (6.5, 6.6)	6.6 (6.5, 6.6)
**MVPA** (minutes/day)	48.2 (35.0, 61.4)	40.8 (34.2, 47.4)
**Dietary intake**		
Discretionary foods (freq/day)	2.4 (2.1, 2.7)	2.3 (2.1, 2.5)
Sugar-sweetened beverages (freq/day)	1.9 (1.6, 2.2)	2.2 (1.9, 2.4)
Takeaway food (freq/week)	3.2 (2.8, 3.6)	3.5 (3.1, 3.9)

BMI: Body mass index; zBMI: BMI z-score; MVPA: moderate-to-vigorous physical activity; freq: frequency.

*Significant $p < 0.05$.

Footnote: Maternal education: low = some secondary education or less; medium = completing secondary school, an apprenticeship or technical certificate; high = university or tertiary qualification. Pubertal status self-assessed using the 'Tanner Stages' [24]. BMI z-score determined using the age- and sex-specific CDC 2000 reference data [18]. Overweight

and obesity determined using the International Obesity Task Force (IOTF) criteria [19].
